# Association Between Beta-Blocker or Statin Drug Use and the Risk of Hemorrhage From Cerebral Cavernous Malformations

**DOI:** 10.1161/STROKEAHA.121.037009

**Published:** 2022-04-13

**Authors:** Susanna M. Zuurbier, Charlotte R. Hickman, Leon A. Rinkel, Rebecca Berg, Ulrich Sure, Rustam Al-Shahi Salman

**Affiliations:** Department of Neurology, Amsterdam University Medical Center, the Netherlands (S.M.Z., L.A.R.).; Edinburgh Medical School, College of Medicine and Veterinary Medicine (C.R.H.), University of Edinburgh, United Kingdom.; Centre for Clinical Brain Sciences and Usher Institute of Population Health Sciences and Informatics (R.A.-S.S.), University of Edinburgh, United Kingdom.; Department of Neurosurgery, University of Duisburg-Essen, Germany (R.B., U.S.).

**Keywords:** adrenergic beta-antagonists, cerebral hemorrhage, hydroxymethylglutaryl-CoA reductase inhibitors, hemangioma, cavernous, central nervous system, secondary prevention

## Abstract

**Background:**

We aimed to determine the association between beta-blocker or statin drug use and the future risk of symptomatic intracranial hemorrhage or persistent/progressive focal neurological deficit from cerebral cavernous malformations (CCM).

**Methods:**

The population-based Scottish Audit of Intracranial Vascular Malformations prospectively identified adults resident in Scotland first diagnosed with CCM during 1999 to 2003 or 2006 to 2010. We compared the association between beta-blocker or statin drug use after first presentation and the occurrence of new intracranial hemorrhage or persistent/progressive focal neurological deficit due to CCM for up to 15 years of prospective follow-up. We confirmed proportional hazards and used survival analysis with multivariable adjustment for age, intracranial hemorrhage at CCM presentation, and brain stem CCM location.

**Results:**

Sixty-three (21%) of 300 adults used beta-blockers (27/63 [43%] used propranolol), and 73 (24%) used statin drugs over 3634 person-years of follow-up. At baseline, the only statistically significant imbalances in prespecified potential confounders were age by statin use and intracranial hemorrhage at presentation by beta-blocker use. Beta-blocker use was associated with a lower risk of new intracranial hemorrhage or persistent/progressive focal neurological deficit (adjusted hazard ratio, 0.09 [95% CI, 0.01–0.66]; *P*=0.018). Statin use was associated with a nonsignificant lower risk of intracranial hemorrhage or persistent/progressive focal neurological deficit (adjusted hazard ratio, 0.37 [95% CI, 0.01–1.07]; *P*=0.067).

**Conclusions:**

Beta-blocker, but not statin, use was associated with a lower risk of intracranial hemorrhage or persistent/progressive focal neurological deficit in patients with CCM.

Cerebral cavernous malformations (CCMs) may present with seizures, intracranial hemorrhage, focal neurological deficit, or may be found incidentally on brain magnetic resonance imaging.^1^ Although neurosurgical excision or stereotactic radiosurgery are performed to reduce the risk of recurrent intracranial hemorrhage, they may cause serious adverse events,^2^ and most patients are managed medically without surgical treatment.^3^ Consequently, the identification of drugs to improve outcome is one of the top 10 priorities for CCM research.^4^

HMG-CoA reductase inhibitors (statins) are of interest as a drug treatment for CCM, aimed at stabilizing the CCM. Statin use might blunt CCM lesion development and intracranial hemorrhage through inhibiting Rho-associated protein kinase. Animal studies and one retrospective cross-sectional study have reported promising outcomes.^5–8^ Beta-adrenoceptor blocking drugs (beta-blockers), specifically propranolol, are also of interest as a drug treatment for CCM.^9^ Beta-blockers might reduce the risk of CCM hemorrhage via antiangiogenic pathophysiological mechanisms, based on in vitro and in vivo findings.^10–12^ There are 2 case reports of the use of propranolol and 2 retrospective cohort studies.^13–16^ The 2 case reports reported good outcomes with propranolol,^15,16^ but the 2 retrospective cohort studies did not find an association between beta-blockers and a decreased risk of intracranial hemorrhage.^13,14^

Therefore, the aim of our study was to investigate the association between the use of beta-blockers or statins (for licensed indications) and the risk of new intracranial hemorrhage or persistent/progressive focal neurological deficit in adults in a prospective, population-based cohort study of CCM.

## Methods

### Data Availability Statement

Data are available upon reasonable request. Data can be shared upon reasonable request for scientific purpose by contacting the corresponding author. De-identified data will be made available to qualified investigators on reasonable request to the corresponding author.

### Study Design and Patient Identification

We included every adult aged 16 years or older, identified by the Scottish Audit of Intracranial Vascular Malformations (SAIVMs), with a first-in-a-lifetime definitive diagnosis of CCM.^17,18^ SAIVMs is an ongoing prospective population-based cohort study of adults who were first diagnosed with any type of intracranial vascular malformation during 1999 to 2003 or 2006 to 2010 while resident in Scotland. We used de-identified data extracts from the SAIVMs database, which did not require specific research ethics committee approval.

### Diagnosis of CCM

First presentation was defined as the symptoms and signs that led to the initial CCM diagnosis. Two neuroradiologists verified CCM diagnoses with reference to accepted criteria^19,20^ and collected data on CCM location and radiological evidence of intracranial hemorrhage.

### Drug Use

We collected data on beta-blockers and statins retrospectively and defined their use as the prescription and receipt of any drug in this class for at least 90 days at any time after CCM diagnosis, but before the first outcome event or the end of follow-up (if an outcome event did not occur). We obtained information from patients’ attendances at secondary care facilities, all of which are within the National Health Service, and from their primary care practitioners who hold a unified record of all correspondence from secondary care, and prescribing records for drugs dispensed by community pharmacies.

### Clinical Outcome and Follow-Up

We obtained data about demographics, medical history, and outcome events from prospective follow-up of hospital records, primary care practitioner records, and postal questionnaires to both the patients and the primary care practitioners, as described elsewhere.^21^ The primary outcome was a composite of new intracranial hemorrhage or persistent/progressive focal neurological deficit definitely attributed to the CCM, as reported elsewhere.^22^ We defined intracranial hemorrhage or persistent/progressive focal neurological deficit according to specific criteria.^1^ Intracranial hemorrhage was defined as a clinical event involving acute or subacute onset of symptoms and radiological evidence of recent hemorrhage consistent with the time of symptom onset. Nonhemorrhagic focal neurological deficit was defined as a new or worsened focal neurological deficit referable to the anatomic location of the CCM without evidence of recent hemorrhage on timely brain imaging. The duration of symptoms was defined as persistent (lasting >24 hours, and staying static or improving) or progressive (lasting >24 hours with further deterioration).^1^ We chose a composite primary outcome of intracranial hemorrhage or persistent/progressive focal neurological deficit because they are of similar severity, and many focal neurological deficits may actually be undetected intracranial hemorrhages.^22^ In patients who had multiple events, the first event was classified as the occurrence of the primary outcome. Intracranial hemorrhage alone was the secondary outcome. We ascertained outcomes using annual prospective surveillance of hospital records, primary care practitioner records, and postal questionnaires to both the patients and their primary care practitioners. Two investigators assessed outcome events using clinical, radiological, or pathological information, masked to drug use. The cause and mode of death were determined using death certificates, autopsy records if post-mortem examination had been performed, or clinical records and pertinent brain imaging studies if death had occurred during a hospital stay. The inception point was the time of first presentation which led to the diagnosis of CCM. We censored follow-up at the first primary outcome, CCM treatment with neurosurgery or stereotactic radiosurgery, or death.

### Standard Protocol Approvals, Registration, and Patient Consents

The Multicentre Research Ethics Committee for Scotland (MREC/98/0/48) and the Fife and Forth Valley Research Ethics Committee (08/S0501/76) approved the observational studies (to which an opt-out consent policy applied) and postal questionnaire studies (which required opt-in consent). The study protocol is available online, www.saivms.scot.nhs.uk/spDesign.asp.

### Statistical Analysis

We performed complete case analyses, and did not impute missing data. We compared baseline characteristics and outcomes between patients with or without beta-blocker or statin use during follow-up. Continuous variables are reported as mean±SD if normally distributed, or as median with interquartile range (IQR). Categorical variables are reported as percentages with their corresponding 95% CIs. For statistical comparisons between 2 groups, we used the χ2 test or, in case of low frequencies, the Fisher exact test. For continuous variables, we used the unpaired *t* test or Mann-Whitney *U* test, as indicated. We used life tables and Kaplan-Meier survival analysis up to 15 years of follow-up, censored at last available follow-up or death unrelated to CCM, followed by multivariable Cox Regression analysis if proportional hazard assumptions were satisfied, with adjustment for the following prespecified covariates because they are potential confounders: mode of CCM presentation (dichotomized as intracranial hemorrhage versus other presentations), location of CCM (dichotomized as brain stem [midbrain, pons, or medulla] versus other locations) and for age because of the baseline imbalance. In another analysis, we also adjusted for antithrombotic drug use after first presentation. We performed a sensitivity analysis of the primary outcome by entering beta-blocker or statin use as a time-dependent covariate. All data were analyzed by SMZ using IBM SPSS Statistics version 25.0.

## Results

A total of 306 adult residents in Scotland were newly diagnosed with a CCM between 1999 and 2003 and 2006 and 2010. After omitting 6 adults who died on the day of presentation and who did not contribute to our outcome analysis, we included 300 adults (Table 1).

**Table 1.**
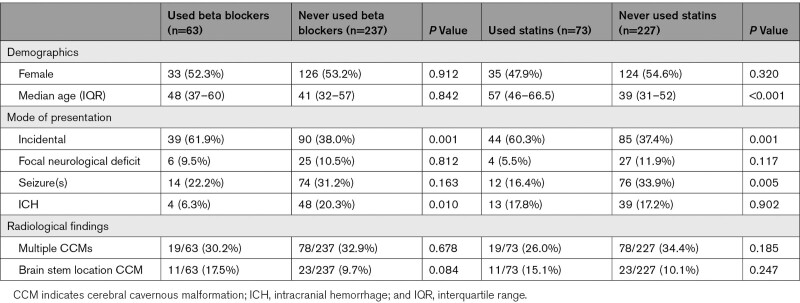
Baseline Characteristics of Adults With a Cerebral Cavernous Malformation, Stratified by Use of Beta-Blocker Drugs and Statin Drugs

Sixty-three (21.0%) adults used a beta-blocker (27/63 [42.9%] used propranolol); 20 of these 63 adults (31.7%) were already using a beta-blocker at the time of CCM diagnosis. The mean duration of beta-blocker use during follow-up was 7.4 years (SD, 5.4 years). The median time to start beta-blocker drug use after CCM diagnosis was 4.0 years (IQR, 1.3–7.8 years). The median duration of beta-blocker use was 3.7 years (IQR, 1.4–8.2 years). The most common indications for the use of beta-blockers were hypertension (28/63 [44.4%]) and migraine prophylaxis (19/63 [30.2%]). Among patients who used beta-blocker drugs, 52.4% were female and the median age was 48 years. The type of presentation was more often incidental (n=39/63 [61.9%] versus 90/237 [38.0%]; *P*=0.001) and less commonly intracranial hemorrhage (4/63 [6.3%] versus 48/237 [20.3%]; *P*=0.01) compared with patients without beta-blocker use. There were no other significant differences in baseline characteristics between both groups.

Seventy-three (24.3%) adults used a statin (26/73 [35.6%] patients used Atorvastatin). Thirty-one of these 73 adults (42.5%) were already using a statin at the time of CCM diagnosis. The mean duration of statin use during follow-up was 7.0 years (SD, 5.1 years). The median time to start statin drug use after CCM diagnosis was 3.0 years (IQR, 0.7–6.4 years). The median duration of statin drug use was 6.9 years (IQR, 2.3–10.3 years). The indication for the use of statins in patients with CCM was cardiovascular or cerebrovascular disease. Among patients who used a statin, 47.9% were female. Statin users were also older, and the type of presentation was more often incidental (n=44/73 [60.3%] versus 85/227 [37.4%]; *P*=0.001) compared with patients not taking statin drugs. There were no other significant differences in baseline characteristics between groups.

Of the 63 patients using beta-blockers, 30 patients also used an antithrombotic drug, 29 patients also used a statin, and 19 patients used a beta-blocker, a statin, and an antithrombotic drug in combination. Of the 73 patients using a statin, 40 patients also used an antithrombotic drug, 29 patients also used a beta-blocker, and 19 patients took all 3 of beta-blocker, statin, and an antithrombotic drug (Table S1).

We followed up the 300 adults with CCM who were alive at initial presentation for the primary outcome of new intracranial hemorrhage or persistent/progressive focal neurological deficit definitely related to CCM for 3634 person-years (of a potential 3843 person-years, for an overall completeness of 95%).^23^ In total, 34 out of 300 patients died during follow-up (11.3%), and their median time of death after CCM diagnosis was 5.1 years.

**Figure 1.**
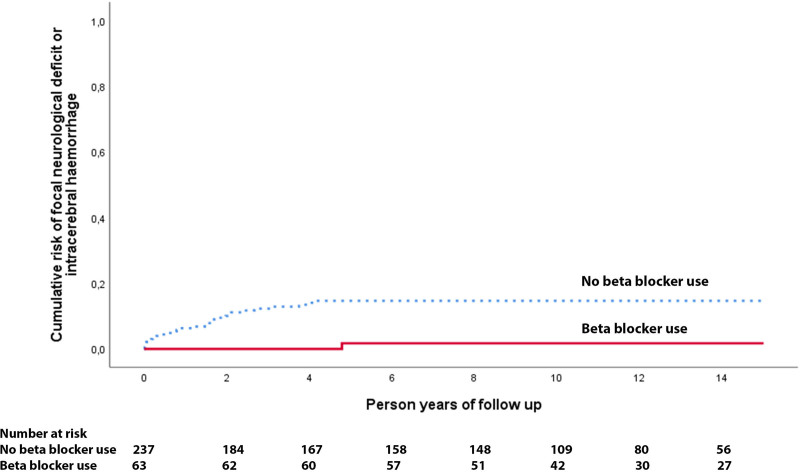
**Kaplan-Meier plot for beta-blockers.** Risk of first intracranial hemorrhage or persistent/progressive focal neurological deficit due to cerebral cavernous malformations according to beta-blocker use during 15 y of follow-up in the Scottish Audit of Intracranial Vascular Malformations.

One of the 63 patients taking beta-blockers developed a primary outcome during 802 person-years of follow-up compared with 29 out of 237 patients not using beta-blockers during 2843 person-years of follow-up (adjusted hazard ratio, 0.09 [95% CI, 0.01–0.66]; *P*=0.018; Figure 1). We were unable to analyze the association between beta-blocker use and a secondary outcome of intracranial hemorrhage alone because there were no outcomes among patients using beta-blockers. In addition, we adjusted for antithrombotic drug use after presentation, resulting in an adjusted hazard ratio of 0.09 (0.01–0.65); *P*=0.017; Table 2. We performed a sensitivity analysis of the primary outcome entering beta-blocker use as a time-dependent covariate, which resulted in a similar direction and magnitude of the association between beta-blocker use and the risk of the primary outcome, but it was no longer statistically significant (adjusted hazard ratio, 0.25 [95% CI, 0.03–1.85]; *P*=0.175).

**Table 2.**
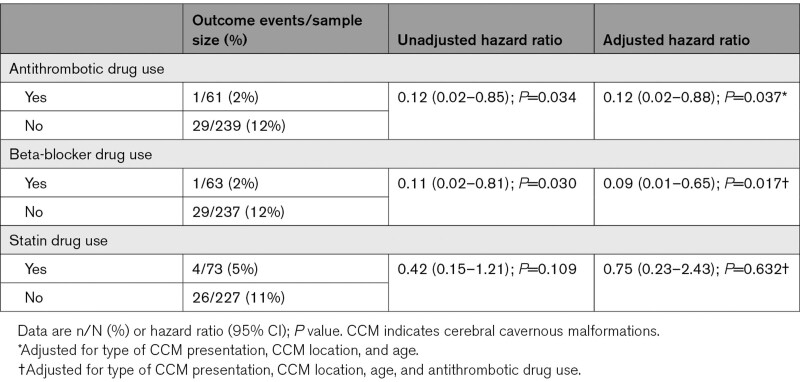
Cox Proportional Hazards Regression of Associations With Intracranial Hemorrhage or Persistent/Progressive Focal Neurological Deficit due to CCM During 15 Year of Follow-Up

**Figure 2.**
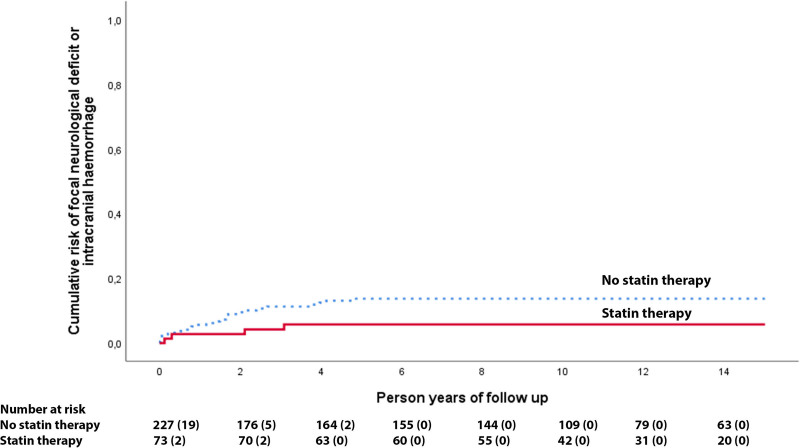
**Kaplan-Meier plot for statins.** Risk of first intracranial hemorrhage or persistent/progressive focal neurological deficit due to cerebral cavernous malformations according to statin use during 15 y of follow-up in the Scottish Audit of Intracranial Vascular Malformations.

Four of the 73 patients on statin drugs developed a new intracranial hemorrhage or persistent/progressive focal neurological deficit during 791 person-years of follow-up compared with 26 out of 227 patients without statin drug use during 2123 person-years of follow-up, which was a nonsignificant reduction (adjusted hazard ratio, 0.37 [95% CI, 0.13–1.07]; *P*=0.067, Figure 2). In a sensitivity analysis, statin use was associated with a nonsignificant reduction in intracranial hemorrhage definitely due to CCM (adjusted hazard ratio, 0.50 [95% CI, 0.14–1.78]; *P*=0.284). For the primary outcome, we additionally adjusted for antithrombotic drug use, resulting in an adjusted hazard ratio of 0.75 (0.23–2.43); *P*=0.632; Table 2. We performed a sensitivity analysis of the primary outcome by entering statin drug use as a time-dependent covariate (adjusted hazard ratio, 0.62 [95% CI, 0.22–1.77]; *P*=0.371).

A post hoc sensitivity analysis revealed similar but nonsignificant associations between beta-blocker (hazard ratio, 0.027 [95% CI, 0.00–723]; *P*=0.488) or statin use (hazard ratio, 0.024 [95% CI, 0.00–432]; *P*=0.456) and the primary outcome when the cohort was restricted to incidental CCMs. Of the 129 patients who presented incidentally, 39 (30%) were treated with beta-blockers (none had a primary outcome) and 90 (70%) were not (3 had a primary outcome). Of the 52 patients who presented with intracranial hemorrhage, 4 (8%) were treated with beta-blockers (none had a primary outcome) and 48 (92%) were not (15 had a primary outcome).

## Discussion

In this prospective, population-based study of adults with CCM, the risk of a new intracranial hemorrhage or persistent/progressive focal neurological deficit in patients using beta-blockers was significantly lower compared with patients without the use of beta-blockers, and this risk was nonsignificantly lower in patients using statins compared with patients not using statins. Beta-blocker (and possibly statin) drug use might be beneficial for the prevention of intracranial hemorrhage or persistent/progressive focal neurological deficit in patients with CCM.

The principal strengths of our study were that we studied an inclusive population-based cohort of adults over a long duration of follow-up. Limitations of our study include its nonrandomized design, recall biases, accuracy of clinical records, and the difficulty of accounting for variable durations of drug use. We did not assess the doses of beta-blocker or statin drugs or adherence nor did we perform genetic analysis to determine which patients with multiple CCMs had an underlying genetic cause.

The initial presentation of CCM was more likely to be incidental and less likely to be with intracranial hemorrhage in patients who took beta-blocker drugs, possibly because beta-blockers may prevent intracranial hemorrhage; because intracranial hemorrhage at CCM presentation increases the risk of recurrent hemorrhage,^24^ we adjusted for this baseline imbalance and the association persisted. Another possible explanation is that patients who took beta-blocker drugs were older and had risk factors for cardiovascular or cerebrovascular disease and are more likely to undergo diagnostic imaging for symptoms/signs unrelated to their CCM but attributable to their underlying cerebrovascular disease. CCM patients who took beta-blocker drugs are also more likely to take antithrombotic drugs for their underlying cardiovascular or cerebrovascular disease, which is another drug class that has been associated with a lower risk of hemorrhage from CCM.^21^ Therefore, we also adjusted for antithrombotic drug use, and the association persisted. Half of the patients on beta-blockers and more than half of the patients on statins also used an antithrombotic drug as secondary prevention for a cardiovascular or cerebrovascular disease (Table S1). However, concurrent use of antithrombotic drugs with beta-blockers or statins does not seem to account for the associations we have observed (Table S1; Table 2) and the paucity of outcomes makes it difficult to establish whether the combined use of these drug classes might be more beneficial than using beta-blockers or statins alone. To reduce the risk of immortal time bias in our study,^25^ we used a time-dependent Cox proportional hazard model, which resulted in a similar direction and magnitude of the associations between beta-blocker or statin drug use and the risk of the primary outcome, but they were not statistically significant.

The association between beta-blocker use and lower risk of new intracranial hemorrhage or nonhemorrhagic persistent/progressive focal neurological deficit in this observational study is promising, although this association is not consistent with a prior retrospective cohort study.^13^

One of the explanations for the nonsignificant reduced risk of intracranial hemorrhage and focal neurological deficit of CCM with statin use could be related to the type of statin used. Most of the patients in our population-based cohort study used simvastatin, whereas atorvastatin reduced CCM hemorrhage in an animal model, while simvastatin did not.^5^ Atorvastatin is twice as potent as simvastatin in measurable effects of Rho-associated protein kinase activity,^26,27^ which might lead to a greater reduction in the risk of intracranial hemorrhage and focal neurological deficit from CCM with atorvastatin. Another possibility is that simvastatin’s effects in animals might not apply to humans: a randomised controlled trial in 10 patients with familial CCM showed no difference in CCM permeability, measured by dynamic contrast-enhanced perfusion magnetic resonance imaging, with simvastatin treatment compared with placebo.^28^ Currently, a proof-of-concept randomised controlled trial of the effect of atorvastatin use for CCM is ongoing.^29^

In conclusion, we found that beta-blocker use was associated with a lower risk of new intracranial hemorrhage or persistent/progressive focal neurological deficit from CCM compared with patients who did not take beta-blockers. Large-scale randomized controlled trials of the efficacy of beta-blockers and statins for the prevention of intracranial hemorrhage from sporadic and familial CCM seem justified, following the completion of ongoing pilot phase trials of the beta-blocker propranolol (Treat_CCM NCT03589014) and atorvastatin (AT CASH EPOC NCT02603328).

## Article Information

### Acknowledgments

We thank Rosemary Anderson, Aidan Hutchison, and all the patients and collaborators in the Scottish Audit of Intracranial Vascular Malformations. The Multicentre Research Ethics Committee for Scotland (MREC/98/0/48) and the Fife and Forth Valley Research Ethics Committee (08/S0501/76) approved the observational studies (to which an opt-out consent policy applied) and postal questionnaire studies (which required opt-in consent). The study protocol is available online, www.saivms.scot.nhs.uk. Dr Zuurbier performed data collection, statistical analysis, and writing of the article. C.R. Hickman performed data collection, statistical analysis, and review of article. Dr Rinkel performed data collection and review of article. Dr Sure reviewed the article. Dr Al-Shahi Salman performed outline of study, data collection, and review of article.

### Sources of Funding

Amsterdam Brain & Cognition Talent Grant, and the Remmert Adriaan Laan Foundation. The sponsors of this study did not take any part in the study design, collection, analysis, and interpretation of data; in the writing of the report; and in the decision to submit this report for publication. The corresponding author had full access to the data in the study and final responsibility for the decision to submit for publication.

### Disclosures

Dr Zuurbier reports grant from Remmert Adriaan Laan Foundation and the Amsterdam Brain and Cognition, during the conduct of the study. No disclosures relevant to this article for C.R. Hickman, Drs Rinkel, Berg, and Sure. Dr Al-Shahi Salman reports collaboration with the Mario Negri Institue for Pharmacological Research for other services and grants from the Medical Research Council, the Chief Scientist Office of the Scottish Government, and The Stroke Association during the conduct of the study; and consultancy fees from BiovelocITA and Recursion Pharmaceuticals, Inc, paid to the University of Edinburgh, outside the submitted work. This work was supported by the Medical Research Council (clinical training fellowship G84/5176, clinician scientist fellowship G108/613, and senior clinical fellowship G1002605).

### Supplemental Material

Table S1

## Supplementary Material


