# Supplementary material for: Association Between Beta-Blocker or Statin Drug Use and the Risk of Hemorrhage From Cerebral Cavernous Malformations
Source: Stroke. 2022 Apr 13;53(8):2521–7. doi: 10.1161/STROKEAHA.121.037009 (PMC9311291; doi:10.1161/STROKEAHA.121.037009)
Supplement: Supplementary file 2 [file str-53-2521-s002.pdf]

SUPPLEMENTAL MATERIAL

Table of contents

Table S1. Overview of medication use and outcome events.

Table S1. Overview of medication use and outcome events.

**Antithrombotic use**	**Statin use**	**Beta-blocker use**	**Primary outcome (ICH or FND)**	**Secondary outcome (ICH)**
yes	no	no	0 (n=10)	0 (n=11)
yes	no	yes	0 (n=11)	0 (n=11)
yes	yes	no	1 (n=21)	1 (n=21)
yes	yes	yes	0 (n=19)	0 (n=19)
no	yes	no	3 (n=23)	2 (n=23)
no	yes	yes	0 (n=10)	0 (n=10)
no	no	no	25 (n=183)	16 (n=182)
no	no	yes	1 (n=23)	0 (n=23)

ICH; intracranial hemorrhage, FND; focal neurological deficit.
